# A novel mutation of COL2A1 in a large Chinese family with avascular necrosis of the femoral head

**DOI:** 10.1186/s12920-021-00995-y

**Published:** 2021-06-04

**Authors:** Zeng Zhang, Kechao Zhu, Huiyong Dai, Qi Wang, Changqing Zhang, Zhenlin Zhang

**Affiliations:** 1 Department of Orthopedic Surgery, Shanghai Jiao Tong University Affiliated the Sixth People’s Hospital, 600 Yi-Shan Rd., Shanghai, 200233 People’s Republic of China; 2 Shanghai Clinical Research Center of Bone Disease, Department of Osteoporosis and Bone Diseases, Shanghai Jiao Tong University Affiliated the Sixth People’s Hospital, 600 Yi-Shan Rd., Shanghai, 200233 People’s Republic of China

**Keywords:** COL2A1, Mutation, Type II collagen, Skeletal dysplasia, Cartilage, Avascular necrosis of the femoral head

## Abstract

Avascular necrosis of the femoral head (ANFH) is a debilitating bone disease, characterized by collapse of the femoral head and subsequent loss of hip joint function. Heterozygous mutations in *COL2A1* have been identified to cause familial ANFH. Here we report on a large Chinese family with ANFH and a novel heterozygous mutation (c.3517 G > A, p.Gly1173Ser) in exon 50 of *COL2A1* in the Gly-X–Y domain. Previously, only five different *COL2A1* mutations have been described in patients with familial ANFH. Therefore, our findings provide significant clues to the phenotype–genotype relationships in familial ANFH and may be helpful in clinical diagnosis. Furthermore, these results should assist further studies of the mechanisms underlying collagen diseases.

## Introduction

Type II collagenopathies represent a group of chondrodysplasias which are expressed as a continuous spectrum of phenotypes, ranging from perinatally lethal (Achondrogenesis II; OMIM 200610) to severe (Spondyloepiphyseal dysplasia congenital; OMIM 183900) to those with only mild arthropathy (Stickler dysplasia; OMIM 108300) [1–4]. The common molecular bases of the type II collagenopathies are heterozygous mutations in the type II collagen gene (*COL2A1*), which encodes the precursor of the type II collagen α1 chain, the most abundant cartilage component [5].

Avascular necrosis of the femoral head (ANFH) is characterized by collapse of the femoral head and subsequent loss of hip joint function. Its clinical manifestations include progressive pain in the groin, pain on exertion, a limping gait, and a discrepancy in leg length. Most cases of ANFH are sporadic, and several etiologic factors (including trauma, alcohol, steroids) have been reported to be implicated [6, 7]. Besides, there are familial cases of ANFH, which may be related to genetic factors. Actually, Liu et al. identified that heterozygous mutations in *COL2A1* caused familial ANFH [8]. Thus, familial ANFH belongs to type II collagenopathies and represents the mild end of spectrum.

However, only five different *COL2A1* mutations have been described in patients with familial ANFH [9–13]. The genotype–phenotype relationship is still poorly understood. Therefore, the studies of more patients with novel mutations in *COL2A1* will be needed for further research to clarify the genotype–phenotype relationship. Here we report one novel mutation in the *COL2A1* gene that causes ANFH in a large Chinese family.

## Materials and methods

### Human subjects

This study was approved by the Ethics Committee of the Shanghai Jiao Tong University Affiliated the Sixth People’s Hospital. All the participants signed informed consent documents according to the Declaration of Helsinki before entering the study.

**Fig. 1:**
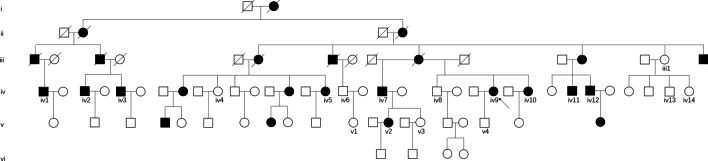
Pedigree of the family. Black symbols indicate affected individuals, and open symbols unaffected individuals

**Fig. 2:**
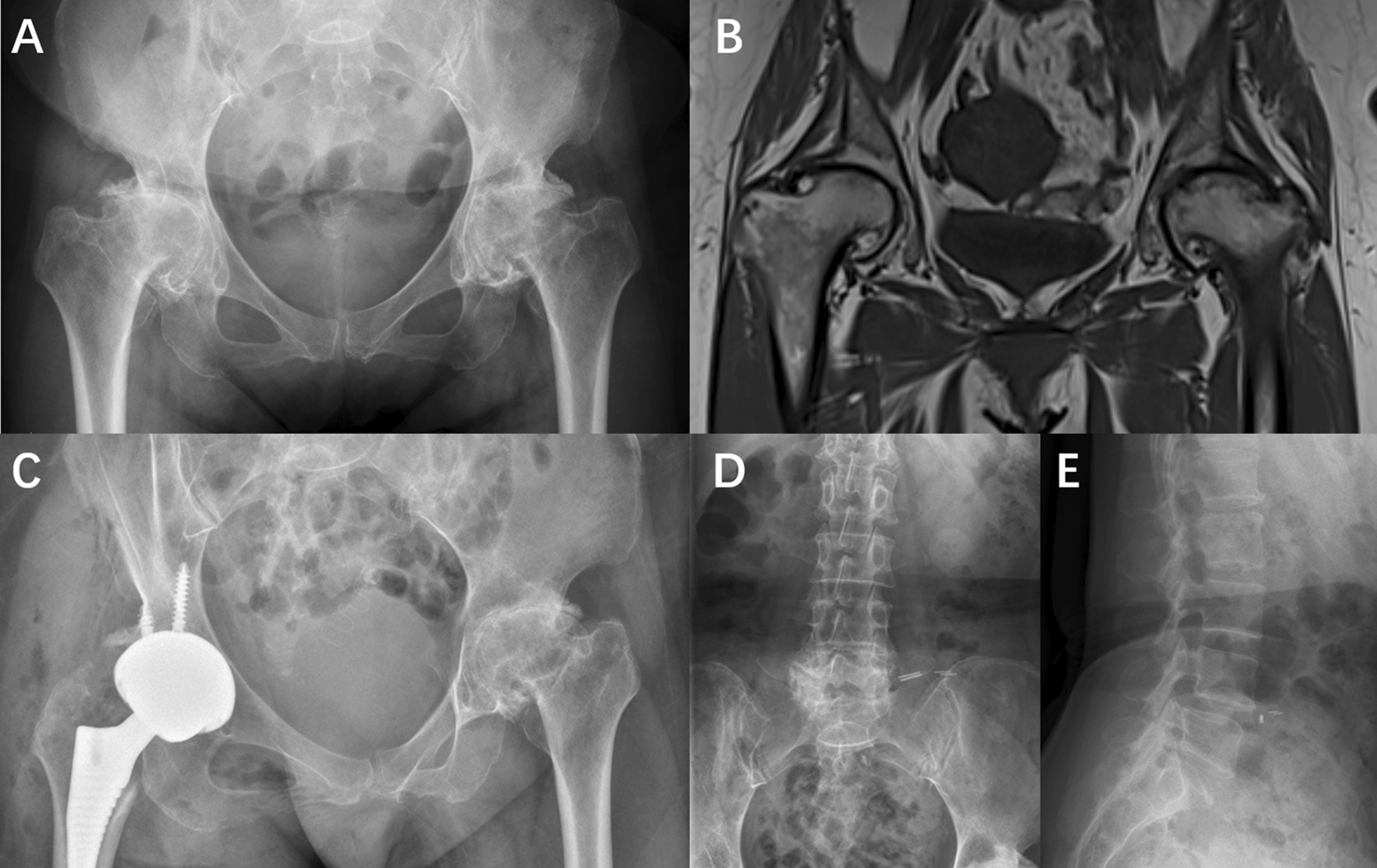
**A**, **B** The X-ray and MRI revealed that collapsed femoral heads with cystic degeneration, and premature hip osteoarthritis in both hips of the proband (IV9). **C** Post-operation radiograph. **D**, **E** The X-ray of the patient's spine indicated normal vertebrae

A large ANFH pedigree (Fig. 1) with a total of 19 subjects was recruited in the present study. Their clinical and genetic information is listed in Table 1. Family members were examined by 2 independent orthopedic surgeons, and imaging results were reviewed by 2 independent radiologists. ANFH were diagnosed using internationally recognized criteria. Age at onset is defined as age at the first appearance of persistent or recurrent limping and/or groin pain, not explained by another cause. The Ficat classification is used to stage avascular necrosis of the femoral head according to plain radiographs, MRI, and clinical features [14]. The proband (IV9), a 51-year-old woman diagnosed with ANFH and premature hip osteoarthritis, visited our hospital due to groin pain and restricted motion of the both hip joints, which started when the patient was 25 years old. A comprehensive survey was conducted to obtain detailed information of the patient's medical history, physical examination and laboratory examination. The X-ray and MRI revealed that collapsed femoral heads with cystic degeneration and premature hip osteoarthritis in both hips, indicating a Ficat stage IV lesion (Fig. 2A, B). The patient was treated with total hip arthroplasty (Fig. 2C). The X-ray of the patient's spine (Fig. 2D, E) was normal and facial features were unremarkable. There were no obvious abnormalities in the patient's neurological system or limbs.

**Table 1:** Characteristics of the family members

	Sex	Age	Disease condition	Ficat classificaion	Age of onset	Height (cm)	Genotype
III1	F	73	Not affected	–	–	166	G/G
IV1	M	49	Affected	IV	36	161	G/A
IV2	M	51	Affected	IV	30	160	G/A
IV3	M	48	Affected	IV	25	163	G/A
IV4	F	58	Not affected	–	–	156	G/G
IV5	F	52	Affected	IV	40	155	G/A
IV6	M	49	Not affected	–	–	180	G/G
IV7	M	62	Affected	IV	40	163	G/A
IV8	M	53	Not affected	–	–	165	G/G
IV9	F	51	Affected	IV	38	157	G/A
IV10	F	49	Affected	IV	20	155	G/A
IV11	M	52	Affected	IV	40	158	G/A
IV12	M	50	Affected	IV	19	160	G/A
IV13	M	50	Not affected	–	–	172	G/G
IV14	F	48	Not affected	–	–	165	G/G
V1	F	25	Not affected	–	–	167	G/G
V2	F	35	Affected	II	26	156	G/A
V3	F	34	Not affected	–	–	159	G/G
V4	M	27	Not affected	–	–	165	G/G

**Fig. 3:**
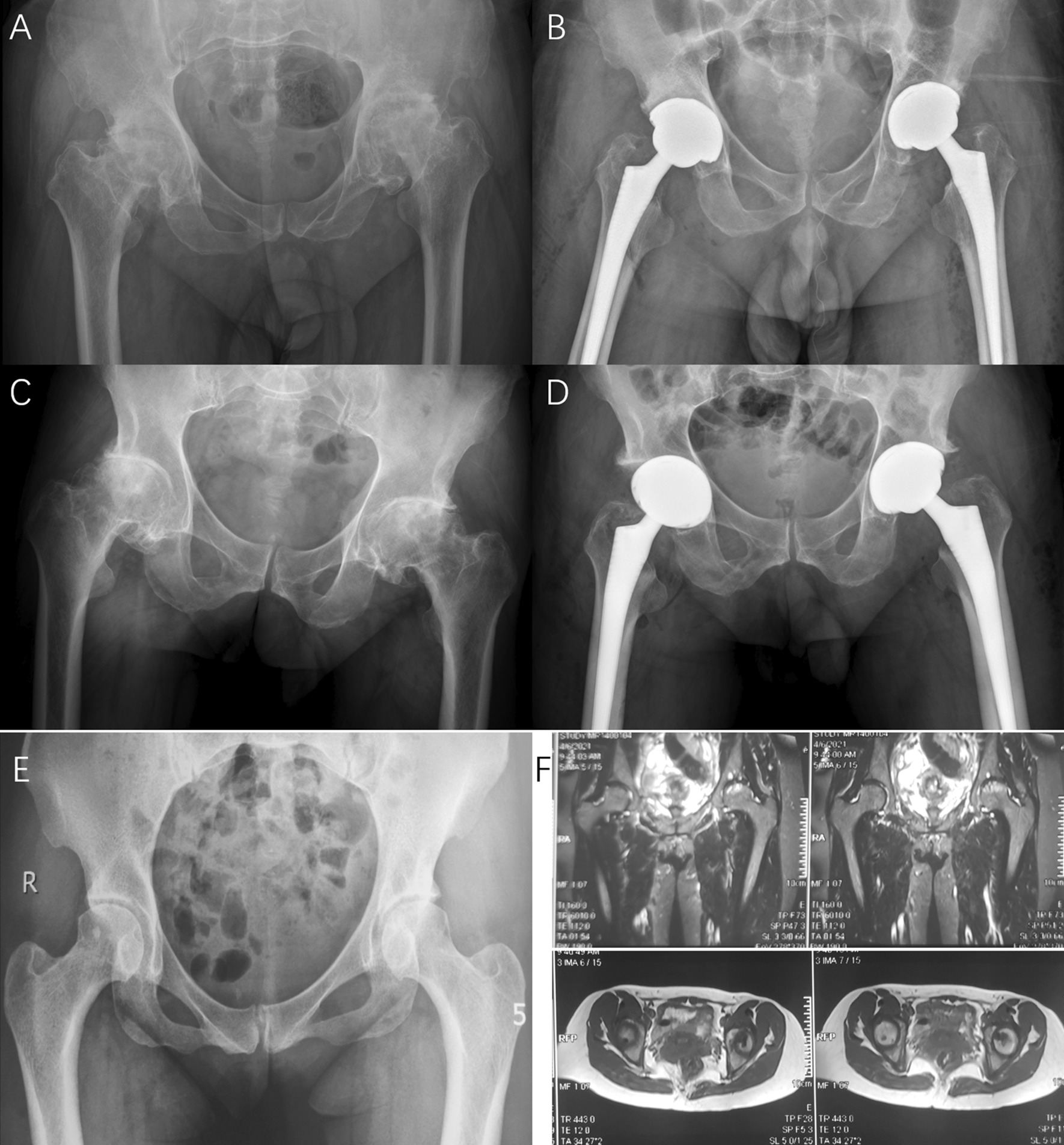
**A**, **B** the pre-operation and post-operation radiographs of IV3. **C**, **D** the pre-operation and post-operation radiographs of IV7. The pre-operation radiographs of both patients (IV3 and IV7) showed severe collapse and deformity of the femoral heads with hip joint space narrowing and osteoarthritis. **E** The radiograph of V2 showed the surface of the femoral head is smooth with no joint space narrowing, but there is localized increasing of bone density and sclerosis at weight-bearing region of the left femoral head. **F** MRI of V2 showed the crescent-shaped hyperintensity region on T2W and lower and uneven signal of necrosis region on T1W in the left femoral head

A total of 10 family members were clinically diagnosed with ANFH and premature hip osteoarthritis. Another two affected family members (IV3 and IV7) also received total hip arthroplasty in our hospital, and the pre-operation and post-operation radiographs were shown in Fig. 3A–D. The pre-operation radiographs also revealed that collapsed femoral heads with cystic degeneration and premature hip osteoarthritis in both hips, which was similar to the proband. It is important to note a relatively young affected family member (V2). This 35-year-old female patient had suffered from groin pain and restricted motion of the left hip joint 10 years ago. Her X-ray (Fig. 3E) showed the surface of the femoral head is smooth with no joint space narrowing, but there is localized increasing of bone density and sclerosis at weight-bearing region of the left femoral head. Her MRI (Fig. 3F) showed the crescent-shaped hyperintensity region on T2W and lower and uneven signal of necrosis region on T1W in the weight-bearing region of the left femoral head, indicating a Ficat stage II lesion.

### Mutation analysis

Informed consent was obtained from the family and from 250 healthy volunteers before blood sampling and DNA analysis. The DNA was extracted from peripheral white blood cells using conventional methods. The DNA sequence for the *COL2A1* gene was obtained from the available online database (GenBank accession No. NC_000012). Primers of the *COL2A1* gene were designed using the Primer 3 software (http://frodo.wi.mit.edu/cgi-bin/primer3/primer3_www.cgi). All exons and their exon–intron boundaries in the *COL2A1* gene were amplified via polymerase chain reaction (PCR). Direct sequencing was performed using the BigDye Terminator Cycle Sequencing Ready Reaction Kit, version 3.1 (Applied Biosystems, Foster, CA, USA), and the sequencing was analyzed with an ABI Prism 3130 automated sequencer.

The interpretation of variants was performed according to the American College of Medical genetics and Genomics (ACMG) guideline [15]. The variants were filtered through the following procedures: (1) the allele frequency of variants is required to be less than 1% or absent from 1000 Genomes Project (The 1000 Genomes Project Consortium 2015). (2) Variants were filtered out when they were synonymous mutation or located in introns without influence on splicing and biological function. PolyPhen-2 (http://genetics.bwh.harvard.edu/pph2/) was used to assess the damaging effects of missense mutations in silico [16].

### Statistical analysis

Continuous variables are presented as means (± SD). A Student’s t test was used to compare the mean values of continuous variables. *p* < 0.05 was considered as significant.

## Results

We recruited a Chinese ANFH family with 10 affected members and 9 unaffected members (Fig. 1 and Table 1). The onset age of the disease in the affected members was 31.4 ± 8.4 years of old. The height of affected male members was 160.8 ± 1.9 cm, while it was 170.5 ± 7.1 cm in unaffected male members (*p* = 0.01). The height of affected female members was 155.8 ± 1.0 cm, while it was 162.3 ± 6.3 cm in unaffected female members (*p* = 0.03). Therefore, both male and female patients in this family have significantly shorter stature than unaffected members.

**Fig. 4:**
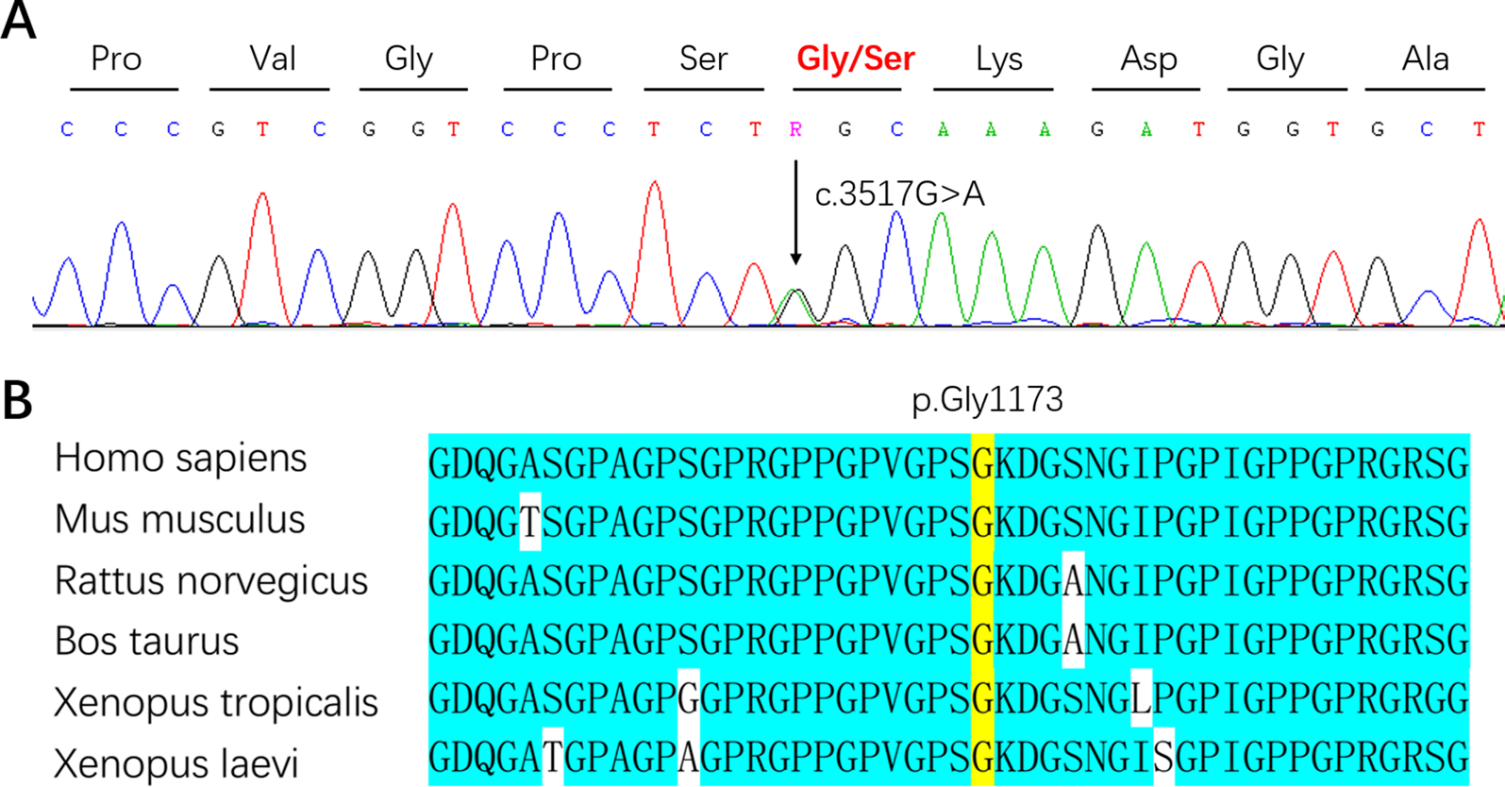
**A** The sequencing revealed a heterozygous 1 bp missense (c.3517G > A) in exon 50, which resulted in p.Gly1173Ser. **B** It is non-conservative, affects evolutionarily highly conserved amino acids from fish to mammals

We screened for the *COL2A1* mutation in the proband using PCR followed by direct sequence analysis. All pathogenic and likely pathogenic variants were manually reviewed according to ACMG guidelines. As a result, we identified a heterozygous 1 bp missense (c.3517G > A) in exon 50, which resulted in p.Gly1173Ser (Fig. 4A). Exon and nucleotide numbering was based on RefSeq NM_001844.4, starting at the ATG translation initiation codon. Sanger sequencing was performed in all available family members (10 affected members and 9 unaffected members) and 250 healthy volunteers. All affected family members carried the heterozygous mutation. It was not present in the unaffected family members, or in 250 healthy volunteers. These results indicated that this mutation was co-segregated in our family and provides strong evidence for the pathogenicity of this mutation. This mutation is non-conservative, affects evolutionarily highly conserved amino acids from fish to mammals (Fig. 4B). It is predicted to be probably damaging with a score of 0.999 by PolyPhen-2 in silico analysis.

## Discussion

The present study identified a novel heterozygous c.3517G > A mutation (p.Gly1173Ser) in the *COL2A1* gene in a large Chinese family. The main clinical characteristics of the affected patients include ANFH and premature hip osteoarthritis, which has been described by the previous studies [10, 11]. In recent decades, > 200 mutations have been identified in the *COL2A1* gene, including single substitution, splice-site mutations, insertions and deletions [1]. *COL2A1* mutations have been associated with various human disorders, which are collectively termed type II collagenopathies [17, 18].

Until now, only six different *COL2A1* mutations have been identified in patients with ANFH. As Table 2 shows, five of the six mutations are glycine to serine substitutions in the Gly-X–Y triple-helix, and Gly1170Ser is the hot spot, which has been identified in four families with ANFH.

**Table 2:** *COL2A1* mutations have been identified in patients with familial ANFH

Protein	cDNA	Region	Race	References
p.Gly582Ser	c.1744G > A	Gly-X–Y	Japanese	Kishiya et al. [19]
p.Gly630Ser	c.1888G > A	Gly-X–Y	Chinese	Li et al. [10]
p.Gly717Ser	c.2149G > A	Gly-X–Y	Chinese	Liu et al. [8]
p.Gly1170Ser	c.3508G > A	Gly-X–Y	Chinese	Liu et al. [8, 9], Su et al. [11] and Wang et al. [13]
**p.Gly1173Ser**	**c.3517G > A**	**Gly-X–Y**	**Chinese**	**The present study**
p.Thr1383Met	c.4148G > A	C-propeptide	Unknown	Kannu et al. [12]

The variants shown are described using the NM_001844.4 transcript reference sequence

Bold indicates *COL2A1* mutation found in this study

The nature of the mutations and their localizations in the protein seem to explain the phenotypic differences, at least to a certain extent [18]. Truncating mutations leading to reduced amounts of normal type II collagen are related with mild phenotypes. In contrast, missense mutations, which replace one Gly residue in the Gly-X–Y repeating pattern, are usually related with severe phenotypes. The Gly-X–Y triple-helix motif is crucial for the proper crosslinking of the pro-α1 peptide chain to form functional type II collagen. Mortier et al. reported that there are numerous excessive post‑translational modifications in type II collagen in patients carrying a Gly-substituted mutation [20].

The exception is glycine to serine substitutions. Glycine to serine substitutions, unlike glycine to nonserine residue substitutions, produced variable phenotypes, with both inter- and intra-familial phenotypic variation [21, 22]. In type I collagenopathies, the severity of the disease has been correlated with the size and charge of the substituted amino acid, specifically Ala < Ser < Cys < Arg < Glu < Asp < Val, in order from least to most disruptive [23]. The same domain-specific effect may exist in type II collagenopathies. Sobetzko et al. identifiedc.3517G > C mutation leading to Gly1173Arg in *COL2A1* in a boy affected with a severe form of spondyloepiphyseal dysplasia [24]. The mutation position is exactly the same with the present study. However, glycine to arginine substitutions usually causes severe phenotypes.

Although most mutations associated with ANFH are glycine to serine substitutions in the Gly-X–Y triple-helix, there is one exception: c.4148G > A (p.Thr1383Met) in the C-propeptide of *COL2A1* gene [12]. C-propeptide mutations typically cause spondyloperipheral dysplasia, characterized by vertebral body abnormalities, hip dysplasia and brachydactyly type E. Therefore, more cases need to be described and more mutations needs to be identified, to clarify the genotype–phenotype relationship.

In summary, we identified a novel heterozygous c.3517G > A mutation (p.Gly1173Ser) in the Gly-X–Y triple-helix motif of *COL2A1* in a large Chinese family with ANFH. Our findings will provide clues to the phenotype–genotype relations and may assist not only in the clinical diagnosis of familial ANFH but also in the interpretation of genetic information used for prenatal diagnosis and genetic counseling.

## Data Availability

The datasets generated during the current study are available in the Mendeley repository, https://doi.org/10.17632/6j2p5t4xns.1.

## Abbreviations

ANFH: Avascular necrosis of the femoral head
COL2A1: Collagen type II alpha 1 chain
SEDC: Spondyloepiphyseal dysplasia congenital
